# Follicular helper T cells (Tfh): heterogeneity in spatial distribution and phenotypic characteristics

**DOI:** 10.3389/fimmu.2025.1686687

**Published:** 2025-10-16

**Authors:** Caifeng Shen, Qiong Fu

**Affiliations:** ^1^ Department of Rheumatology, Renji Hospital, Shanghai Jiao Tong University School of Medicine, Shanghai, China; ^2^ Shanghai Immune Therapy Institute, Shanghai, China

**Keywords:** Tfh, germinal center, follicular mantle, circulating Tfh, Tfh plasticity, Tfh1, Tfh2, Tfh17

## Abstract

Follicular helper T cells (Tfh) are a Th cell subset that directly assists B cells in functioning, and their development is regulated by various factors. Among them, the initial regulation leads to phenotypic heterogeneity, while the regulation of their migration process results in spatial heterogeneity. The phenotypic heterogeneity is manifested by the presence of Tfh subsets with characteristics helper T cells (Th) of other lineages, namely Tfh1, Tfh2, and Tfh17, with different transcriptional programs and secrete distinct cytokines, potentially possessing different functions. The spatial heterogeneity is mainly manifested by the positional relationship between Tfh and germinal centers (GC), which are mainly divided into GC-Tfh, follicular mantle Tfh, and circulating Tfh, possibly reflecting the process of Tfh occurrence. This review summarizes the spatial and phenotypic heterogeneity of Tfh cells, and suggests a Tfh cell type framework with nodes of previous studied cell types and the edges of switching between specific celltypes, which is affected by the summation of imprinted plasticity part and *de novo* plasticity part in Tfh development, connecting the hypothesis Crotty et al. proposed in 2018. Discrete cell type is still eligible in qualifying the diseases state and quantifying the activity and severity of diseases, but it could also be beneficial to look Tfh from the view of cell states and expression programs, which, in the future studies, might better model the through process of Tfh development and unifying the contradiction caused by separate Tfh cell type view.

## 1 Introduction

Follicular helper T (Tfh) cells are a specialized CD4+ T helper subset of adaptive humoral immunity. Orchestrated by the lineage-defining transcription factor BCL6(B-cell lymphoma 6), Tfh cells are primed in the T cell zone and undergo full differentiation within GCs (germinal centers) of secondary lymphoid organs, where they provide critical signals for B cell selection, antibody affinity maturation, and class-switch recombination through direct cell-contact and cytokine production (e.g., IL-21) (1).

A defining hallmark of Tfh cells is their profound heterogeneity, which can be categorized along two primary dimensions: spatial distribution and functional phenotype. Based on their anatomical positioning, Tfh cells are compartmentalized into distinct subsets: GC-Tfh residing within GCs, FM-Tfh (follicular mantle Tfh) in FM (follicular mantle zone), cTfh (circulating Tfh) patrolling the periphery blood, and peripheral helper T cells (Tph) (2), as well as mucosal tissue Tfh. They share B cells helper function, although they possess differences in phenotype. Tfh cells exhibit functional plasticity, giving rise to subsets that co-express the typical transcription factors and cytokines of other T helper cell lineages (e.g., T-bet(T-box Transcription Factor, TBX21)/IFN-γ in Tfh1, GATA3/IL-4 in Tfh2, and RORγT(RAR-related orphan receptor C)/IL-17 in Tfh17), thereby tailoring antibody responses to specific immunological challenges.

Pathological conditions, including infections, autoimmune disorders, and cancers, often feature significant perturbations in the abundance, subset composition, and function of Tfh cells and their relatives, underscoring their pivotal role in disease pathogenesis. However, studying these relationships is complicated by the dynamic and plastic nature of these cells. This review aims to synthesize current understanding of Tfh cell heterogeneity, integrating spatial, phenotypic, and functional perspectives. We will explore the developmental pathways, migration dynamics, and diverse roles of canonical Tfh subsets, while also dedicating focused discussion to the emerging roles of Tph cells and Tfh/Tfh-like populations in mucosal immunity.

### 1.1 A multi-stage journey: Tfh cell differentiation, migration, and maturation​

The differentiation of Tfh cells is a tightly regulated, multi-stage process that can be broadly delineated into initiation, migration, and effector phases, culminating in their functional specialization within GCs (3). Tfh cell fate is primed at the T-B border of secondary lymphoid organs. Here, naive CD4+ T cells receive critical signals from antigen-presenting cells (APCs) (primarily dendritic cells (DCs)), including MHC-II complexes, costimulation molecules, and cytokine signaling (4–6). This synergistic signaling cascade activates BCL6, which defines the Tfh lineage. BCL6 orchestrates Tfh commitment by driving the expression of canonical Tfh markers like CXCR5 and repressing genes that promote alternative T helper cell fates (e.g., T-bet/Th1, GATA3/Th2, RORγT/Th17) and the Tfh antagonist BLIMP1 (7, 8), making naive CD4 polarize towards Tfh rather than non-Tfh T helper cells (4, 9). Additional transcription factors, including BATF (Basic-Leucine Zipper Transcription Factors), ASCL2 (Achaete-scute family bHLH transcription factor 2), c-Maf (Musculoaponeurotic Fibrosarcoma Protein), and act upstream or in concert with BCL6 to reinforce this developmental program and promote IL-21 production (10–13).

Upon upregulating CXCR5 and downregulating the T-zone homing receptor CCR7, newly committed pre-Tfh cells migrate towards the CXCL13 chemokine gradient into the follicle and mature into GC-Tfh (14–16). The initial positioning of pre-Tfh within GC is regulated by a balance of CXCR5, CCR7, and EBI2 signaling (17). Within GCs, sustained interactions with APC (particularly through ICOSL (Inducible T-cell costimulator Ligand), CD40 by B cells, and CD115/CD112 by DCs) are indispensable for the final maturation of GC-Tfh cells (18–20). Mature GC-Tfh cells are characterized by high expression of BCL6, CXCR5, PD-1, and ICOS, and are proficient secretors of IL-21, which is essential for supporting B cell differentiation and antibody production (3). By downregulating CCR7 (17) and responding to CCL19/CCL21 signal (16), pre-Tfh could also migrate into FM to mature into FM-Tfh.

**Figure 1:**
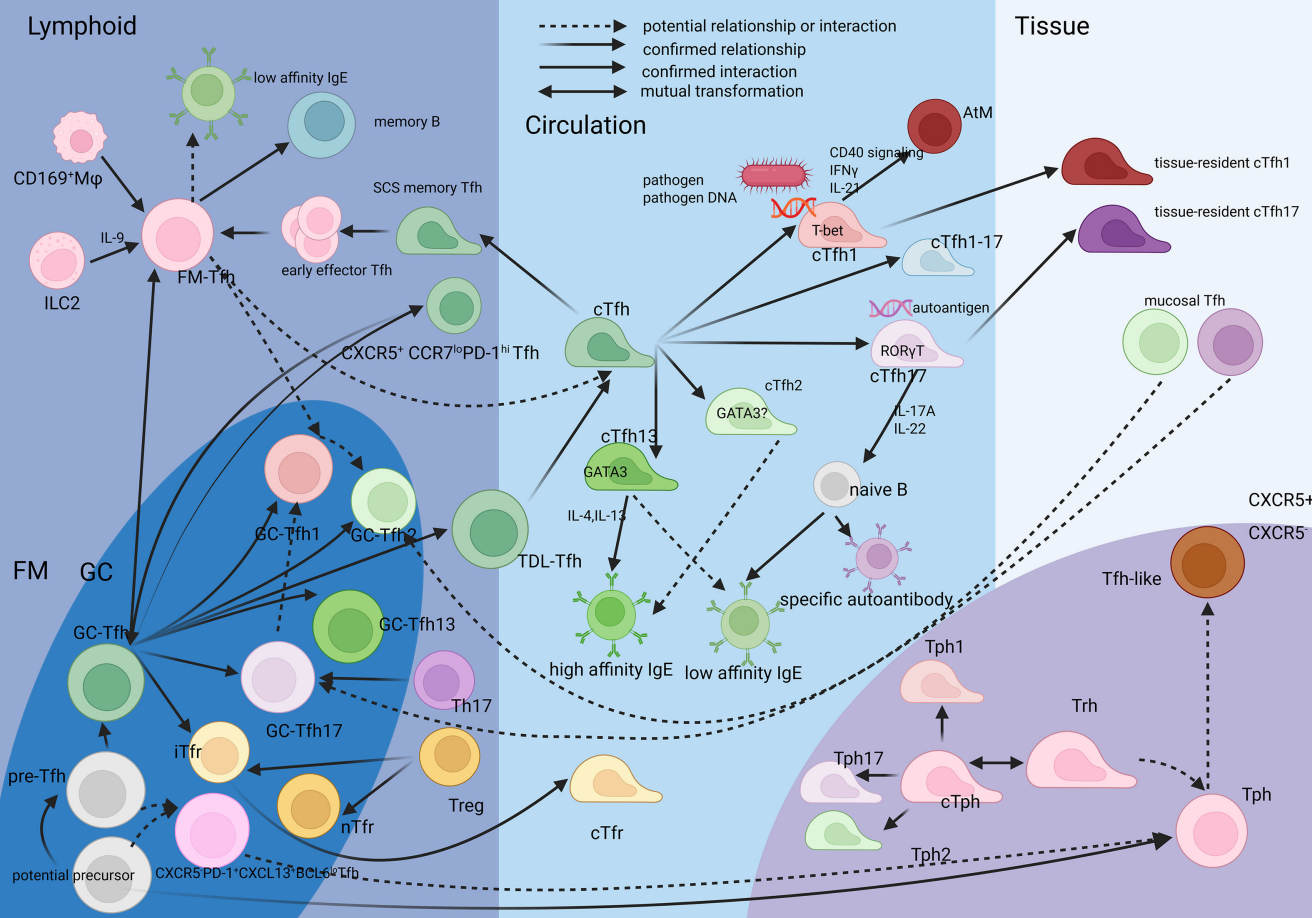
The relationship between the Tfh, Tph and their spatio and lineage subsets FM-Tfh is a stable subset different from pre-Tfh and GC-Tfh, while it is generated from GC-Tfh and pre-Tfh. cTfh is considered originated from GC-Tfh and also FM-Tfh. The initiation milleus is deduced to influence their phenotype. cTfh1 and cTfh17 can migrate into pathogenic tissue. Most mucosal Tfh has a Tfh17 skewing. Tph and Tfh have a common precursor closer than Th22, Tph has itsr circulating counterpart like cTfh, also relevant to memort phenotype.Tfh-like cells are considered relevent to Tph. Created in BioRender. SHEN, C. (2025) https://BioRender.com/jah121c, with permission.

GC-Tfh could form a transitional CXCR5^+^CCR7^lo^PD-1^hi^ migrating subset which traverses the subcapsular sinus into efferent lymphatics, migrating toward the thoracic duct with a phenotype converging with circulating Tfh (cTfh) (21) by CD69, CD90, Ki-67 downregulation, and KLF2(Kruppel-like factor), S1PR1(Sphingosine-1-Phosphate Receptor 1), S1PR2(Sphingosine-1-Phosphate Receptor 2) upregulation (22, 23). Moreover, thoracic duct Tfh (TDL-Tfh) retain the transcriptional and epigenetic imprints of GC-Tfh, their TCR clonotypes are enriched in peripheral cTfh, confirming the migration route and the phenotype variation from GC-Tfh to TDL-Tfh and then to cTfh (23), which can be further proven by the clonal overlap between cTfh and GC-Tfh and their shared feature of expressing BCL6 and IL-21 (24, 25) (**Figure 1**).

### 1.2 Tfr: hybrid regulators of germinal center dynamics​​

Follicular regulatory T cells (Tfr) serve as a pivotal immunoregulatory subset within GCs, co-expressing BCL6 (shared with Tfh cells) and FOXP3 (characteristic of Tregs), alongside a hybrid phenotype combining Tfh-like surface markers CXCR5^+^PD-1^+^ICOS^+^ and Treg-like surface markers CTLA-4^+^GITR^+.^ The Tfr derived from thymic Tregs (CD25^hi^CD38^+^) with closer resemblance to Treg TCR repertoires are called natural Tfrs (nTfrs), they localize to GC mantle/dark zones, and their limited TCR diversity support their role in suppressing autoreactive GC B cells clones via their preference in autoantigen, assisted by suppressive molecules (e.g., CTLA4), neuritin (preventing GC B expansion), and decoy receptor IL-1R2/IL-1R antagonist(preventing GC B activation) (26, 27).

Beyond direct B cell targeting, Tfr also modulates the GC microenvironment to prevent excessive immune responses. The timing of naive T cells’ entry into the GC influences their differentiation due to the microenvironment of GCs: early-arriving precursors exposed to strong TCR and costimulatory signals tend to adopt a Tfh phenotype, whereas late-arriving cells—encountering declining antigen availability, reduced IL-6/IL-21, and enhanced inhibitory cues (e.g., CTLA-4, IL-10)—preferentially differentiate into Tfr (28), which is referred to as induced Tfrs (iTfrs) and considered to originate from CD25^hi^Tfh precursors under IL-2 signaling and GC microenvironment cues. They operate predominantly in germinal center light zones, where they modulate GC contraction by suppressing excessive Tfh responses while providing help to affinity-matured GC-B (e.g., via IL-21 and IL-10 secretion) (29, 30) to maintain their survival in dark zone microenvironments while inhibiting their activation (26, 28). Moreover, “late Tfr”, which enters GCs late, is a subset of iTfr, which induces GC B apoptosis via prolonged interactions (likely by FasL signal) (31). It mediates GC termination and could also be considered as immune suppression. Therefore, it can be hypothesized that the functional polarization of Tfr cells is influenced by their developmental proximity to either Tfh or Treg lineages, implying that the transition from Treg or Tfh to Tfr may represent a functional and phenotypic continuum shaped by both cellular origin and microenvironmental cues (**Figure 1**).

Systemic immune surveillance involves circulating Tfrs (cTfrs, BCL6^+^CXCR5^+^CD4^+^FOXP3^+^CD25^+^), which are hypothesized to patrol between blood and lymphoid tissues via S1P and CXCR5-CXCL13 axis and home to nascent GCs to prime regulatory networks. This subset is considered as “immature Tfr”: it exhibits reduced immune suppression and may accelerate secondary antibody responses (32); it has a lower expression of BCL6 and Tfh-like surface markers than Tfh, while maintaining a similar expression of FOXP3 with Treg and lower suppressive function than GC-Tfr (33). Anti-PD-1 therapy could increase cTfr, consequently increasing the quantity of plasmablasts and the titer of immunoglobulin, both of which exhibit positive linear correlation with CD38^+^ cTfr (34) (might be considered as “i-cTfr” subset), suggesting a deviation from traditional Tfr function.

To conclude, Tfh biology is governed by multilayered control: Transcriptional antagonism (BCL6 vs. BLIMP1), cellular positioning guided by spatiotemporal molecular concentration gradients, and functional cross-talk with Tfr. Defining these subsets necessitates integrating phenotypic markers, functional outputs, and anatomic context to resolve their roles in immunity and autoimmunity.

## 2 Heterogeneity of canonical Tfh cells: GC-Tfh, FM-Tfh, cTfh​​

As outlined in the introduction, Tfh heterogeneity is defined by spatial and phenotypic criteria. This section will now detail the characteristics of the canonical spatial subsets: GC-Tfh, FM-Tfh, and cTfh.

### 2.1 Germinal center Tfh

GC-Tfh cells, characterized by high BCL6, PD-1, CXCR5 expression and a signature of CD44^hi^CD62L^lo^CD90^lo^CCR7^lo^PSGL1^lo^CXCR5^hi^ICOS^hi^PD-1^hi^ (16), are not a monolithic population but exhibit remarkable functional plasticity, driven by disparate cytokine milieus and transcriptional programs that confer T helper lineage-like characteristics (Tfh1, Tfh2, Tfh17). This heterogeneity is empirically supported by *in situ* multiplex immunofluorescence and CyTOF analyses (35).

#### 2.1.1 Distribution-specificity and lineage subset characteristics of GC-Tfh

The abundance and activation state of GC-Tfh cells vary significantly across secondary lymphoid organs, reflecting their distinct immunological roles. Flow cytometry reveals a quantitative gradient in Tfh frequency of tonsil>mesenteric lymph nodes (mLNs)>spleen and activation markers (e.g., Ki-67, CXCR5, PD-1, ICOS, HLA-DR, CD38) of tonsil > spleen > mLN.Tfr populations mirrored this activation gradient (tonsil > spleen > mLN) for marker expression but demonstrated an inverse frequency distribution pattern of being enriched in mLNs compared to tonsils or spleen (36). This suggests tonsils are active reaction centers, the spleen is a storage site, and mLNs may function as regulated transit hubs, consistent with their roles in antigen trafficking. Despite these quantitative differences, the qualitative properties and defining features of Tfh lineage subsets skewing remain comparable across these sites, allowing for generalized subset classification.

**Figure 2:**
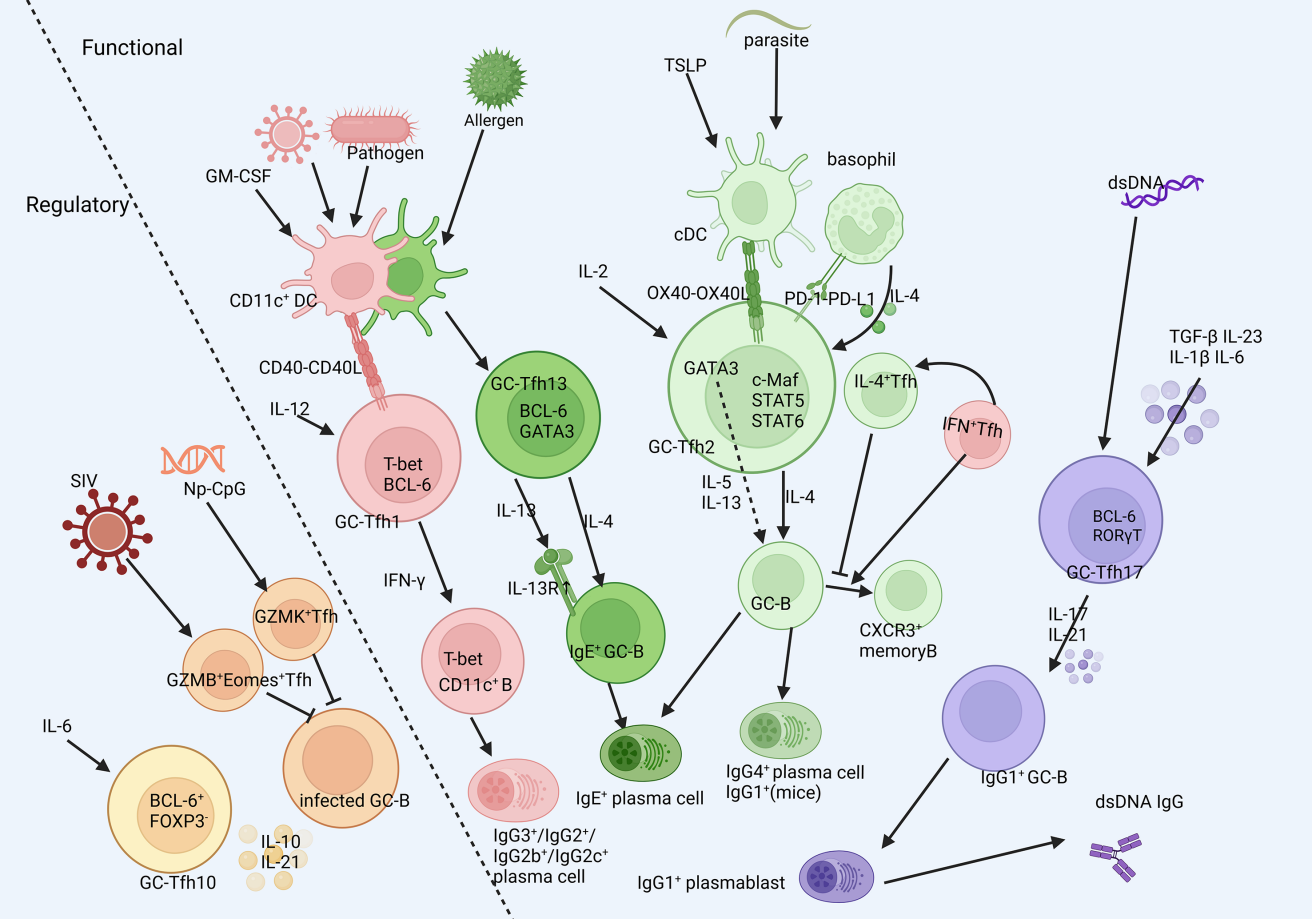
The heterogenity of GC-Tfh cells. GC-Tfh could be classified into several subsets different in functions according to their surface markers and classical transcription factors and cytokines. They are initiated in different milleus, experience deviate developmental process and respond to different stimulation. Created in BioRender. SHEN, C. (2025) https://BioRender.com/cej3yjb, with permission.

GC-Tfh skewing is highly context-dependent (**Figure 2**). Under homeostasis, it exhibits limited heterogeneity and similar transcriptional profiles. However, during disease disturbance, the APCs (mainly DCs) and cytokine milieu potently reshape GC-Tfh polarization, prompting the expression of transcription factors associated with other Th lineages and secretion of related cytokines (37–39), thereby tailoring antibody responses to specific immunological challenges. Their differentiation is initiated by specific cues from antigen-presenting cells (APCs), predominantly dendritic cells (DCs), which shape the ensuing polarization. The characteristics of the major subsets are summarized in **Table 1**.

**Table 1:** Major GC-Tfh lineage subsets.

Subset	Key inducing signals	Master regulator	STAT pathway	Effector cytokines	Primary functions & pathological associations
GC-Tfh1	CD40L(by cDC) (40), IL-12 (41–43)	T-bet (41)	STAT3 (6, 43),STAT4 (42, 43),STAT1 (5)	IFN-γ (41)	•influences GC formation and the expansion/maintenance of GC-B cells within it (44) •Promotes class-switching of human IgG2(virus and its vaccination)/IgG3(intracellular protozoa) and mice IgG2b(virus)/IgG2c(Rickettsia, virus)/IgG3(virus) (44–48) •Helps naïve B cells differentiation into AtM (42, 45, 49) •Critical for anti-intracellular pathogen immunity.
GC-Tfh2	IL-2 (5), IL-4 (from basophils) (50), OX40L ​​(from DCs) (51), PD-L1(from basophils) (50)	GATA3(not necessarily),c-Maf, CNS2 (52–55)	STAT5,STAT6 (5)	IL-4 (52–55)	•Drives class switch of IgE(allergy/helminth) and IgG1(mice)/IgG4(human) production (56–59) • Central to anti-helminth responses.
GC-Tfh13	(less defined)	GATA3 (60)	(less defined)	IL-4, IL-5, IL-13 (60)	• Critical for IgG1^+^ B cell conversion to IgE^+^ plasma cells(IL-4) and driving high-affinity IgE(IL-13) in allergic responses (60). • Positively correlates with disease severity in asthma and food allergy (61).
GC-Tfh17	IL-6, IL-1β, IL-23, TGFβ (62–64)	RORγT/IRF4/BATF (65–68)	STAT3/STAT4 (63, 64)	IL-17 (65–67), IL-17R, IL-23R (63)	•Enhance IgG1+ plasmablast differentiation and IgG1 secretion (63) •Correlates with auto antibodies and autoimmune disease severity (63)

​​Beyond these main subsets, regulatory subsets also exist in GC-Tfh, including Tfh10 and cytotoxic Tfh. Tfh10 shares features with conventional Tfh but produces high levels of IL-10 while lacking Foxp3 and CD25, distinguishing them from Tfr. They sense inflammation (e.g., via IL-6) and feedback with IL-10 to establish an anti-inflammatory balance (69). Cytotoxic Tfh cells highly and specifically express GZMK (granzyme K) and GZMA (granzyme A), and also GZMB (granzyme B). They can kill GC B cells to suppress high-affinity antibody production or eliminate infected cells, but may also disrupt GC homeostasis if overactivated. They appear more closely related to the Tfh1 subset and play a role in chronic inflammatory and fibrotic diseases (70–72).

#### 2.1.2 Issues in GC-Tfh subtyping​​

Traditionally, Tfh subsets were classified by cytokine profiles or transcription factors, but both methods present limitations. There are misalignments of cytokines and transcription factor. It has been proved that GC-Tfh2 relies on c-Maf and CNS2 rather than GATA3(which is low in Tfh2); the generation of IL-5/IL-13 strictly depends on GATA3, and IL-4^+^ Tfh itself does not co-express IL-5 or IL-13 (52–55). Also, some argue that IL-4 expression does not necessarily confer a Th2-biased heterogeneity to GC-Tfh (73), yet in parasite models, these IL-4^+^ Tfh do promote host IgE and IgG1 responses against helminths (74). Additionally, the more Th2-like Tfh13 subset (producing IL-4/5/13 and GATA3-dependent) better qualifies as “bona fide” GC-Tfh2 than IL-4-only producers. Therefore, it seems as if the commonly referred to “IL-4^+^ GC-Tfh2” is not an exact classification, and its heterogeneity warrants specific investigation. Similarly, IL-17 production in GC-Tfh17 is not strictly RORγT-dependent but is ICOS-dependent. In the absence of RORγT, IL-6/IL-23 signaling could activate STAT3, which cooperates with IRF4/BATF to bind the IL-17A promoter and thus sustain IL-17A production (68), indicating that GC-Tfh17 generation is a process coordinated by multiple transcription factors and cytokines. Thus, cytokine and transcription factor signatures often misalign, underscoring the need for a more precise and dynamic classification system.

Surface marker-based classification used for cTfh (e.g., CXCR3/CCR6) also fails to fully align with cytokine/transcription factor subset, suggesting the subpopulations defined in GC-Tfh and cTfh may not be entirely analogous. Simultaneous analysis using CXCR3/CCR6 markers and cytokines (IFN-γ, IL-17) found some of the GC-Tfh subset(e.g., Tfh1 and Tfh17) identified by surface markers(CXCR3/CCR6) without coexpression of IL-21 and their corresponding cytokines, but this misaligned fraction of cells in marker-identified GC-Tfh1/GC-Tfh17 are decreased as the increase of corresponding environmental stimuli, suggesting the “aligned” GC-Tfh might be considered as an activated state. Interestingly, there actually exist Tfh1/17 cells (CXCR3^+^CCR6^+^) producing IL-17, IL-21, and IFN-γ simultaneously, although they are rare in the CXCR3^+^CCR6^+^CXCR5^+^ subset, where IL-17/IFN-γ single-expressing subsets dominate (37). Single-cell RNA sequencing also revealed cross-polarization in a particular environment: ~8% of Tfh under Tfh1-enriched conditions exhibited Tfh2 traits, and ~15% under Tfh2 conditions showed Tfh1 features (72). These discoveries suggest the misalignment of surface markers and cytokines might also largely derive from the impact of environmental stimuli and their corresponding state, other than the inaccuracy of certain markers.

### 2.2 Tfh in follicular mantle zone is distinct from pre-Tfh and GC-Tfh

Follicular mantle zone(FM), the area surrounding the GC, is localized between the GC and the capsule of follicles. It is enriched by IgD^+^ naive B cells, and thus could be distinguished from GC by CD157 or CD21(both positive in GC) and IgD(positive in FM) markers. And by technique based on IgD^+^naive B cell enrichment and Tfh migration velocity gradients (lower migration velocity in FM), the span of the FM zone is measured to be about 50 μm (21, 75). FM-Tfh denotes a spatially defined functional subset that stably occupies the FM and scans antigens via subcapsular sinus (SCS) macrophages (16, 21), while having shared but a little lower expression of surface molecule features CXCR5, PD-1, and ICOS than their GC counterpart (GC-Tfh) (76) (**Figure 1**).

Approximately 30% of FM-Tfh cells differentiate directly from pre-Tfh without traversing GCs, indicating a GC-independent developmental pathway (77). This process is orchestrated through BCL6-mediated regulation of PSGL1, EBI2, and S1PR1 genes, which disrupts the barrier of exchange Tfh between GC and FM (78), and correspondingly, transformation could happen between FM-Tfh and GC-Tfh (21), thus FM-Tfh serves as one of the sources of GC-Tfh.

However, despite this exchange, FM-Tfh presents different molecular profile, shaping their function deviated from GC-Tfh under resting state: While expressing *Bcl6* transcript levels comparable to GC-Tfh, they exhibit significantly reduced BCL6 protein expression (~60% decrease) (21), which stems from IL-9 receptor (IL-9R) signaling sustaining baseline BCL6 expression, as evidenced by further BCL6 downregulation upon IL-9 deprivation (79), suggesting their low polarization state. Cytokine secretion profiles demonstrate elevated IL-10 production but diminished IL-4 output compared to GC-Tfh (21). The IL-10 secretion inhibits memory B cell apoptosis, complementing GC-Tfh-driven proliferation, both of which are vital in memory pool maintenance. Under disturbance such as LCMV infection, FM-Tfh can express T-bet and IFN-γ, acquiring Th1-like effector functions (80), in which STAT3 is also vital (6). This plasticity is facilitated by persistent IL-9R expression, enabling responses to IL-9 secreted by ILC2s activated via B cell-derived leukotriene LTC4 (79). Critically, an intermediate KLF2 expression gradients dictate spatial specificity of FM-Tfh and renders them susceptible to perforin-dependent NK cell clearance, which is also counteracted by IL-9 signaling (79, 81), suggesting IL-9 might be an indispensable factor in FM-Tfh development and regulation.

The transcriptional reprogramming of FM-Tfh resembles GC-Tfh profiles during rechallenge, accelerating antibody production rates five-fold versus primary responses (21). FM-Tfh gain access to antigens from CD169^+^ macrophages, which enables efficient presentation to memory B cells, reactivating secondary GC reactions (25). Thus, FM-Tfh may function as a multifunctional subset that orchestrates the interplay between innate immunity and GC/EF(extrafollicular) reactions, maintains immune stability at steady state, and mounts rapid responses upon antigen re-encounter.

Pathologically, FM-Tfh causes allergy while avoiding autoimmune responses and presents age-association. Constrained by the absence of somatic hypermutation(SHM) in follicular mantle B cells, FM-Tfh could only induce IgE^+^B cells, without generating autoreactive IgE antibodies (60), and eventually induce low affinity IgE, which play a role in allergy, due to their rapid antibody generation and reaction, for example, IL-9R^+^FM-Tfh drive five-fold increases in house dust mite (HDM)-specific IgE, with IL-9R blockade significantly ameliorating allergic responses (79). On the contrary, since GC-Tfh experiences SHM, high-affinity antibodies could be induced, for example, GC-Tfh13 are capable of inducing the differentiation of B cells into plasma cells producing high-affinity IgE (60). Accumulation of FM-Tfh (increasing by 40%) and the gradual decrease of GC-Tfh both correlate with age and impaired GC reactions, explaining reduced vaccine efficacy in elderly populations (21, 76). Therefore, it could be deduced that recalibrating FM-Tfh/GC-Tfh balance by targeting KLF2-S1PR1 or IL-9R signaling might potentially offer a novel intervention for allergic disorders and age-related immune decline.

FM-Tfh is rarely studied due to the difficulties in dissecting (owing to the localization and the overlap phenotype with GC-Tfh), and the pathological role is less significant than GC-Tfh and cTfh, while studying it might give an insight of the potential relationship between GC-Tfh and cTfh. Real-time visualization of pre-Tfh, FM-Tfh, and GC-Tfh trafficking is important in directly observing their relationship; and studying the crosstalk of KLF2 and IL-9R(including whether KLF2 directly suppresses *Il9r* transcription to regulate FM-Tfh expansion) and the consistency of KLF2^int^ IL-9R^+^ co-expression in human lymph nodes versus tonsils could further decode the development of FM-Tfh and deepen the insight into their roles in Tfh plasticity.

### 2.3 Circulating follicular helper T cell

Circulating follicular helper T cells (cTfh) are characterized by the surface markers CXCR5^+^CD45RA^−^CD45RO^+^. Although considered to originate from GC-Tfh, cTfh exhibit reduced BCL6 expression alongside elevated CCR7 levels and moderately decreased CXCR5 expression (77, 82–84). Their function alterations are associated with phenotypic regulation and spatial localization. Besides acting as a long-lived memory pool, cTfh is activated upon re-stimulation and undergoes clonal expansion (32, 77, 85) to stimulate B cell differentiation for antibody production. They play vital roles in anti-viral neutralizing antibody production (39, 86), while the abnormal increase of cTfh correlates with autoimmunity (87). They facilitate the differentiation of naïve and memory B cells into plasmablasts secreting IgG, IgE, and IgA via T–B interactions (84), mainly depending on their secretion of IL-21 (88, 89). This process is antigen-specific, with variations in quantity and effector function. For instance, following influenza, tetanus, or *Candida albicans* vaccination, MHC-II peptide tetramer technology allows isolation of cTfh specific for each antigen (90). Intriguingly, cTfh cells are more potent than effector Tfh cells in lymph nodes: they can be reactivated by DCs, home to GCs, and produce more cytokines (23), aligning with their memory function and re-activation under stimuli.

#### 2.3.1 Circulating Tfh with memory phenotype

cTfh has four developmental states: naïve (Tn), stem-like memory (Tscm), central memory (Tcm), and effector memory (Tem). Finally, cTfh can mainly display either Tem or Tcm phenotypes (91). Upon initial generation, cTfh exhibit a Tem phenotype (low CCR7), consistent with GC-Tfh and later gradually transition to a Tcm state (high CCR7) following antigen stimulation (83, 92, 93), to be a predominate relatively stable memory subset in homeostasis conditions, it adopts a more “stem-like” phenotype, including intrinsically expression of CCR7 and CD62L mediating migration (93) and a lower expression of transcription factors and cytokines of Tfh lineage subset than in cTfh-Tem, whereas Tfh-Tem are less frequent (94), and expand abnormally under specific diseases (95), probably due to a dominant effect in the corresponding milieu (91). The extent of cTfh memory states activity is determined by CCR7(stem-like markers), ICOS, and PD-1(activation markers). PD-1^++^CCR7^lo^ cells rapidly differentiate into mature Tfh upon antigen re-encountering (77). Among these, ICOS^+^PD-1^++^CCR7^lo^ cells (<1% of cTfh) express Ki-67, representing activated memory cTfh, whereas ICOS^−^PD-1^+^CCR7^int^ and ICOS^−^PD-1^−^CCR7^hi^ subsets correspond to resting memory cTfh (96). Shifts toward activated Tem phenotypes are clinically significant; for example, RA patients exhibit increased frequencies of Tfh-Tem (PD-1^hi^CCR7^lo^/ICOS^+^PD-1^hi^CCR7^lo^), which correlate with disease activity (95, 97), indicating antigen-driven activation. Thus, the acquisition of an activated Tem phenotype (characterized by PD-1^hi^CCR7^lo^) brings cTfh closer both phenotypically and functionally to GC-Tfh, suggesting that cTfh become active because of the transformation of their memory state.

#### 2.3.2 Heterogeneity of cTfh

Blood cTfh is categorized into three functional subsets based on surface chemokine receptors analogous to Th lineages: cTfh1 (CXCR3^+^CCR6^-^), cTfh17 (CXCR3^-^CCR6^+^), and cTfh2 (CXCR3^-^CCR6^-^) (96). A rare cTfh1–17 subset (CXCR5^+^CXCR3^+^CCR6^+^) has also been identified and implicated in B-cell responses (28, 98), though its scarcity has limited mechanistic insight. cTfh2 secrete IL-4, IL-5, and IL-13 but not IL-6 or IL-10, distinguishing them from Th2 cells by their lack of anti-inflammatory function. cTfh1 secrete IFN-γ and express T-bet, while cTfh17 secrete IL-17A and IL-22 and express RORγT (84, 99), resembling Th1 and Th17 profiles, respectively (**Figure 1**).

During viral infections,cTfh1 expand and promote virus-specific IgG production by assisting memory—but not naïve—B cells, underscoring a specific helper phenotype (99–101). Pathogen-associated DNA-induced cTfh stimuli further induce IFN-γ^+^ cTfh (102), reinforcing their role in antiviral immunity. cTfh1 also exhibits pathogenic potential: they provide CD40L, IFN-γ, and IL-21 to promote CD21^lo^T-bet^hi^ with CD40 activation outweighing TLR signaling (103). CXCR3 mediates cTfh1 migration to inflammatory sites such as the CSF in multiple sclerosis, where they display a cytotoxic gene signature (104). Tfh1 are found in lupus nephritis kidneys (105)and IgA vasculitis intestine (106). and their cTfh1 reduction in peripheral blood suggests tissue homing.

cTfh17 can produce IL-21 and help naive B cells (84). They are strongly implicated in autoimmunity: about 60% of autoreactive cTfh are IL-17 positive and exclusively express RORγT and drive specific autoantibody induction by memory B cells in a RORγT-dependent manner, thus qualitatively and quantitatively associated with autoimmune disease (102, 107). Furthermore, beyond their helper function, cTfh17 cells also present expansion in serum and target tissue infiltration, such as the kidneys of IgAV nephritis and the aorta of Takayasu arteritis (108, 109). However, CCR6 is not Tfh17-specific (73), and BCL6 itself can suppress CCR6 expression (78),complicating subset identification.

cTfh2 also produces IL-21, but its specific functions remain unclear. Their defining markers (CXCR3^-^CCR6^-^) are negative, and the previously assumed key cytokine IL-4 is not specific to cTfh2. Classification by CXCR3 and CCR6 double negativity includes a significant amount of non-GATA-3/non-IL-4 expressing cells (56), suggesting CXCR3/CCR6 double negative does not fully characterize cTfh2. Some studies have begun using CXCR3^-^CCR4^+^ markers for cTfh2 (37, 110, 111). Functionally, cTfh2(referred to as GATA-3^+^IL-4^+^BCL6^+^CXCR3^-^CCR6^-^) promotes B cell differentiation into antigen-secreting cells and positively correlates with total and antigen-specific IgE and allergic reactions (112). It also participates in autoimmune, considering its positive correlation with SLEDAI and anti-dsDNA antibodies in SLE (56). Furthermore, an IL-13-producing cTfh13 subset is elevated in peanut allergy and asthma, correlated with IgE, and airway hyperreactivity (60), suggesting the crucial role of cTfh13 in high-affinity IgE in allergy, consistent with GC-Tfh13 (113), calling for studies on the relationship of cTfh2 and cTfh13.

Notably, distinct cTfh subsets (e.g., cTfh1/cTfh17) may adopt different memory stages according to the immune milieu; in healthy individuals, cTfh1 primarily exibits Tem and Tcm is enriched in cTfh17, and in viral infection cTfh1 signatures effector state while cTfh17 still in memory state (91), suggesting the role of cTfh1 in normal secondary re-challenging, by sustaining a more active state within memory state. It is different in autoimmune diseases, such as active SLE, in which cTfh1 primarily exhibits Tcm, while cTfh17 shows a Tem phenotype (114), consistent with the effect of Tfh17 skewing. Thus, cTfh subsets enter Tem state to exert their function of different lineages in various conditions.

### 2.4 Plasticity of GC-Tfh and cTfh

Heterogeneous Tfh phenotypes are stimulus-dependent. Crotty proposed three models—’imprinted partial plasticity,’ ‘*de novo* partial plasticity,’ and ‘FM-Tfh origin’—to explain the mechanisms underlying cTfh heterogeneity (73), which, as we noticed in recent researches, could be borrowed to model the heterogeneity and plasticity of all Tfh subsets. These plasticity models operate within the established framework of the BCL6-BLIMP1 balancing axis (section 1.1), often starting from a low-polarity state (84). *In vitro*, under IL-12/IL-4/TGF-β conditions, memory-like induced-Tfh1/2/17(iTfh) can be generated, respectively. These cytokine-induced Tfh1/2/17 express Tfh core markers and “canonical markers” for each Tfh lineage, with BCL6 lower than in GC-Tfh but comparable to pre-Tfh and cTfh (91), supporting the idea that decreased BCL6 inhibition contributes to Tfh subset generation (13, 64). and their effect in antibody class switching is consistent with the corresponding lineage, suggesting the existence of a relatively stable state in Tfh skewing (91), supporting the concept of low-polarity Tfh and environment-driven polarization (“*de novo* partial plasticity”). By downregulating RORγT and IL-17 while upregulating IL-21 and BCL6, Th17 cells in Peyer’s patches(PP) can transform into Tfh-like cells (115). Gut segmented filamentous bacteria(SFB) could also generate Tfh17 by inducing c-Maf rather than BCL-6; these Tfh17 share origin and TCR profile with Th17 rather than Tfh. S1PR1+ Tfh17 enter circulation and migrate to the spleen, potentially contributing to cTfh17. Aligned with cTfh17 effects we described above, these splenic Tfh17 promote RA manifestation, enhance GC-B responses, and elevate anti-GPI antibody titer (116), supporting the “imprinted” plasticity models, and its adjustment of c-Maf suggests that BCL-6 is not the only factor of tuning Tfh and Th lineage phenotype, and considering c-Maf being the key of GC-Tfh2 generation, supporting the imprinted plasticity model applied to GC-Tfh, where c-Maf is an important participant, as a supplement to its *de novo* plasticity model (5) supported by evidences in 2.1.2 and 2.1.3.

These models help explain plasticity from bona fide Tfh to lineage subsets, but direct evidence for plasticity between established Tfh subsets is still scarce, with most data limited to subset ratio change (117), rather than the direct evidence of transition between the “individual” Tfh lineage subset cells. But it is certain that there might be a shared period between Tfh1 and Tfh2 in their early stage. GC-Tfh1 transiently express T-bet during early maturation and undergo epigenetic modifications at the *Ifng* locus, enabling sustained IFN-γ production (41), and surprisingly, so-called “GC-Tfh2” cells can transiently produce IFN-γ before switching to IL-4, which could be referred to as an IL-4-free window, which is a transition regulated by T-bet (51, 58). IFN-γ production promotes the differentiation of germinal center B cells (GC-B) into CXCR3^+^ memory B cell precursors. When secretion switches to IL-4, this program gradually downregulates to modulate GC dynamics (118), suggesting that the early-stage-GC-Tfh1 actually has the same function as “mature” Tfh1. These could be interpreted to assume the potential transformation from (transient) Tfh1 to Tfh2; the possibility of plasticity might lie in the misalignment of Tfh subset markers. It has been indicated that Tfh2 only secretes IL-4 in helminth infection to help low-affinity IgE generation, and Tfh13 in allergen stimulation helps high-affinity IgE generation (60). As Tfh13 co-expresses IL-4 and IL-13, it might be hypothesized that Tfh2 could transfer to Tfh13. Another potential evidence of potential plasticity between Tfh lineage subsets is that under autoimmune uveitis(AE), the expression of decreasing or deficient STING (Stimulator of interferon genes) could drive Tfh17 polarization, and in the infiltrated tissue, the proportion of Th1 is decreased; however, in the DLN of AE, the Th1 is high (119). Given that STING is the driver of IRF3, IFN-I and IFN-γ receptor expression and Th1 differentiation (120), despite the necessity of direct “Tfh1 to Tfh17” evidence, it could be deduced that the polarization of Tfh17 could also be a consequence of Tfh17 skewing overcoming Tfh1 skewing. Additionally, the presence of Tfh1–17 cells (37) might also hint at a potential connection between Tfh1 and Tfh17 subsets, which could be worth exploring. There is no hint of the transformation between Tfh lineage subsets after their skewing is mature, suggesting that upon maturation, Tfh subsets enter a relatively stable state, and their relatively pliable state exists prior to this stable state.

## 3 Non-canonical Tfh-like populations​​

The conventional definition of Tfh cells—based on the co-expression of BCL6, CXCR5, and PD-1—fails to capture the full spectrum of CD4+ T cells capable of providing B cell help. Emerging evidence has revealed populations of non-canonical Tfh-like cells that, while lacking one or more of these classic markers (most notably CXCR5), nonetheless exhibit core Tfh functional attributes and a potent capacity to drive B cell differentiation and antibody production in peripheral and mucosal tissues. The recognition and classification of these non-canonical populations are essential for a complete understanding of humoral immunity, particularly in pathological contexts where they often play prominent roles.

### 3.1 Peripheral helper T cells

Peripheral helper T cells (Tph) are a distinct CD4+ T cell subset with a CXCR5^-^PD-1^+^CXCR3^+^CXCL13^+^IL-21^+^IL-10^+^BLIMP1^hi^BCL6^lo^ signature (121). They are originally identified in rheumatoid arthritis (RA) synovium, and have a unique capacity to drive memory B differentiation into plasma cells in extralymphoid tissues (2). A hallmark of Tph differentiation is the TGFβ and ICOS-driven transcriptional program via Sox4, which promotes CXCL13/IL-21 secretion, PD-1 upregulation, and downregulates CXCR5, simultaneously enabling their homing to inflammatory sites via alternative chemokine receptors (e.g., CCR2/CCR5/CX3CR1) while precluding their entry into B cell follicles (122–125). While sharing a dependency on c-Maf, BATF, TIGIT, etc. with Tfh cells, their low BCL-6 and inability to access B cell follicles due to CXCR5 deficiency represent key distinctions (2, 62, 126, 127).

Tph cells exhibit significant plasticity and contextual function, while originally upregulating IFN-γ secretion (124) and acquiring Tfh1-like features when driven by combined ICOS and TGFβ signaling (2, 127). Upon acute viral infection, Tph upregulates CXCR3, TBX21, and STAT1, produces IFN-γ, to induce CXCR3 expression and neutralizing antibodies of plasmablasts (121). In chronic inflammation and autoimmunity, they are pivotal drivers of pathogenic antibody production by stimulating memory B cells to differentiate into plasmablasts and pathogenic antibody, especially IgG production in infiltrated tissues (125, 128), including synovium(RA) (128) and kidneys(SLE) (127). Similar to GC-Tfh1 and cTfh1, Tph can also promote CD21^low^CD11c^+^ B cell differentiation, also by offering IFN-γ, CD40 and IL-21 (129). All these evidences suggest that Tph has a Th1 skewing like Tfh1, thus it might also be recognized as “Tph1”. But it could also be possible that those Tph studied in autoimmune disease are originally generated under Interferon milieus, for it is observed that by blocking IFNAR, Tph and CXCL13 generation can be halted (130). Besides, a spectrum of Tph subsets (Tph1, Tph2, Tph17, Tph1-17) exists in the circulation pool of Tph(referred to as circulating Tph, cTph), with the dominant subset influencing disease manifestation. They are also classified using CXCR3/CCR6. Among them, Tph2 and Tph1–17 subsets expressed only low level of IL21 and offer less help to B cells; Tph2 presents a cytotoxicity related transcripts correlated with their alignment of severity and activity of autoimmune disease (131); SLE patients exhibit increased Tph1/Tph2 frequencies positively linked to SLEDAI scores, with Tph1 enrichment in cutaneous/musculoskeletal involvement and Tph2 in lupus nephritis (132), suggesting the different roles Tph subsets play across the system of SLE. Interestingly, these Tph2 upregulate T-bet, Eomes, etc, indicating the participation of Tfh1-skewing programs. However, there are also evidence that Tph2 expand in allergic disease, secreting IL-5, IL-13, IL-21, likely to induce IgE-producing B cells (45, 112), different from that in autoimmunity (131), thus Tph2 might be heterogeneous, although it is necessary to clarify whether this heterogeneous is “imprinted” or from the stimulation of milieu in “*de novo*” development.

The relationship between Tph and Tfh is complex and may represent a continuum rather than a strict dichotomy. Beyond shared differentiation mechanisms and some common signatures mentioned previously, Tph and Tfh might also share the same developing pathway: IL-2–STAT5 signaling negatively regulates both lineages, while aryl hydrocarbon receptor (AHR) synergizes with AP-1(Activator Protein-1) family member JUN(Jun proto-oncogene) to inhibit CXCL13^+^ Tph/Tfh differentiation and promote Th22 development (130), so it could be deduced that at least, at the time point T helper cell develops Th22, Tph and Tfh are still sharing the same trajectory, and Tph/Tfh could be considered as subsets with shared mechanism but possess separate fate at last, probably under the effect of different milieus, the IFN-γ fate overcome the AHR/JUN signal. Besides, a distinct population of CXCR5^-^PD-1^+^CXCL13^+^BCL6^lo^ Tph-like Tfh cells within breast cancer TLS promotes robust B cell recruitment and TLS formation, and might present a phenotypically “in-between” subset between Tfh and Tph (133). Moreover, although with a low expression, BCL6 is vital in Tfh and most Tph generation(although exceptions exist in lung resident Tph) and correlates with auto-antibody generation (134), again indicating the closer relationship of Tph with Tfh than other T helper cells.

cTph are homologous to pathological tissue resident Tph with shared features, functions, and, most importantly, TCR repertoire with cTfh cells (134), again suggesting deep correlation of Tfh and Tph. There is also evidence that cTph comes from and could in turn become tissue resident T helper cells(Trh) by adjusting their CD69 and PSGL1 levels, and due to their shared phenotype and highly overlapped TCR clonal type, the Trh could be considered as Tph reside in affected tissue. Trh can be distinguished from cTph by a CD69^+^PSGL1^lo^ pattern (135). The Tph in synovium also highly expresses CD69, CXCR6 as Trm(tissue resident memory T helper cells) markers (136), suggesting that Trh might be a Tph compartment partially overlapped with Trm, BLIMP-1, and GPR56 participate in tissue residency (136, 137) (**Figure 1**). More importantly, although the IL-21R expression is lower in Trh and cTph, the IL-21-IL-21R-BCL6 signaling axis is still vital in their development (135), suggesting their strong relationship with Tfh as a tissue resident compartment.

In total, it is previously considered that Tph is a stable subset apart from Tfh as they have different differentiation track, but the transcriptome and experimental researches argues that Tph might also be regarded as a cell state of “generalized” Tfh, so Tph and Tfh in blood and pathogenic tissues might have redundant functions, but due to the difficulty of recruiting Tfh in chronic inflammatory tissues like synovium, their amount overwhelms Tfh (2) Furthermore, given that Tph strongly produce CXCL13, and CXCL13 can recruit Tfh and B cells by CXCR5-CXCL13 axis we discussed previously, Tph might be the initiator of autoimmune. As a result, quantitative comparison of Tfh and Tph B cell helping efficiency will further reveal the significance of recruiting Tfh, either as an enhancer of building ectopic GC or just recruited from the circulating system unspecifically. However the findings of tumor-infiltrated Tph that do not secrete IL-21 but instead recruit Breg cells by providing TGF-β and PD-L1 challenges this unified view (138), these cells may not have the same origin with Tph in inflammary tissues, but it is also rational to reason that it is within the tumor microenvironment that Tph changes its state or become a new subset, and it could be valuable to compare this Tph with “bona-fide” Tph on the a larger panel of Tph signatures, tracking how Tph enter these pathogenic tissues may be significant in further illustrating Tfh and Tph relationship.

### 3.2 Canonical and non-canonical Tfh in mucosal tissues

Tfh cells across various mucosal sites typically retain the canonical CXCR5+BCL6+ phenotype but exhibit tissue-specific functional biases. Significant conceptual and phenotypic challenge arises from the discovery of CXCR5^-^ cells that closely mimic key features of Tfh cells, termed Tfh-like cells. They occur in mucosal tissues and might play a role in pathological mucosal immunity. In this part, we will focus on these mucosal Tfh populations.

#### 3.2.1 Tfh and Tfh-like cells in GALT

As referred to at 2.1.1, the Tfh in tonsil, spleen and mLN are comparable despite quantitative differences, but Tfh in Peyer’s patches(PP) presents a Th17 bias apart from integrin, whereas the core program is the same; these could be considered as another evidence for the effect of imprinted plasticity (73). The Tfr in PP express BATF and c-Maf and secrete IL-4 and IL-21, a small group of Tfr also express IFN-γ under the regulation of T-bet (139), presenting a Th1 skewing. These evidences suggest an intrinsic skewing milieu in PP.

It was previously thought that GC-Tfh17 in GALT induces IgA production (115). However, recent findings argue that intestinal IgA does not depend on Th17 or IL-17. TGF-β produced by Peyer’s patch Tregs is the primary driver of IgA production, and IL-21 produced by Tfh is essential for terminal differentiation of IgA plasma cells; both factors are indispensable. These IgA-inducing Tfh may originate directly from naïve T cells and do not appear to exhibit a polarized phenotype (140, 141).

IgE production is also regulated in GALT. Food antigen could induce IL-4 producing Tfh(referred to as Tfh2) in mesenteric lymph node and Peyer’s patches, CD40 provided by DCs and ICOSL provided by B cells together with their presenting antigen induce and maintain this Tfh in germ-free mice, which is associated with IgE producing plasma cells generating and IgE increasing (142), suggesting the actual IgE-promoting effect of Tfh2 and the importance of gut microbiota. Moreover, peanut-specific cTfh13 is sensitively decreased in peanut oral immunotherapy and might help decrease IgE, although it does not necessarily predict the success of this therapy (143). Together, it is suggested that IgA rather than IgE is the most important factor in oral immunization.

There exist Tfh-like cells characterized by their CCR9^+^CXCR5^-^ phenotype in GALT. They share a strikingly similar transcriptional profile with bona fide Tfh cells, including elevated expression of BCL6, c-Maf, PD-1 and ICOS, and retain the capacity to produce IL-21. These cells primarily localize to mucosal sites, especially the gut-associated lymphoid tissue (GALT), reflecting the role of CCR9 in gut homing. Thus, according to its residency and markers, it would not be too courageous to infer that CCR9^+^Tfh-like cells are the Tph counterpart in GALT, especially in the large picture of inflammatory and autoimmune states. They could also display remarkable functional plasticity, producing high levels of diverse cytokines such as IFN-γ, IL-17, IL-4, and IL-10 (144), in response to complex microenvironmental cues. Consequently, mounting evidence implicates these CCR9^+^ Tfh-like cells in the pathogenesis of autoimmune conditions. They contribute to mucosal inflammation in inflammatory bowel disease (IBD) (likely via IL-17/IFN-γ driven pathways) (145) and participate in the exocrine gland damage seen in primary Sjögren’s syndrome (pSS) (potentially through ectopic IL-21 production supporting autoreactive B cells) (146), all of which indicates that Tfh-like cells might have a close relationship, or at least emerge in very similar state with Tph.

#### 3.2.2 Tfh-like cells in BALT

It has recently been revealed that ectopic inducible bronchus-associated tissues (iBALT) in the lung formed in inflammation and infection have similar ectopic GC-like structures, which could be distinguished from typical GC or other ectopic GC by a vague T-B aggregation structure. In viral and *Mycobacterium tuberculosis* infection, iBALT-Tfh has the canonical GC-Tfh phenotype (147), particularly, in tuberculosis background this Tfh represent a Tfh1 skewing with a CXCR3^+^T-bet^+^IFN-γ^+^ phenotype (148), while in G^-^ bactorial infection background, a non-canonical “Tfh-like” cell without CXCR5, but with a normal PD-1, could offer help to GC-B-like cells with CD40L, IL-21, and IL-4, interestingly this non-canonical subset shows a valid Th2 skewing, with GATA3 expression and IL-10, IL-13 secretion (149).

#### 3.2.3 Tfh-like cells in NALT

Although sharing a canonical GC-Tfh phenotype, the frequency of NALT(Nasopharyngeal-associated lymphoreticular tissue) Tfh is declined in adults, compared to those in children, which might be associated with the degradation of the adenoid. Inactivated antigen vaccine and LAIV(Live-attenuated influenza vaccines) could induce the proliferation of NALT Tfh, the generation of GC-B cells and the enhancement of the antibody production in NALT. In addition, the adjuvant CpG-DNA could enhance Tfh and antibody responses, which might have potential linkages with Tfh17 biased phenotype (150, 151).

In conclusion, Tfh have their counterpart in mucosal tissues, according to the milieus, these Tfh presents lineage-skewing, and the Tfh cells (particularly in GALT/NALT) is age-associated, suggesting that they might be temporal subsets that mainly functions in the environment of immature immunology; while the CXCR5^-^ Tfh-like cells might be considered as a unique Tfh subset owing to their B cell helping function, it could be proposed that these “Tfh-like” cells might be Tph counterpart in mucosal, they loss CXCR5 possibly due to the lack of typical GC or ectopic GC-structures in mucosal, and they are not very prominent in producing CXCL13, which might be owed to the way B cells enter the corresponding mucosal structure is quite different from which in normal GC and normal ectopic GCs, these reason might also explain the lack of CXCR5 in Tph, suggesting that Tph and Tfh are “counterpart” in different milieus. Ultimately, it might be the environmental context that endow Tph with different lineage phenotypes or gene expression programs and signatures.

## 4 Discussion​​

The differentiation of Tfh cells is governed by a complex and precise regulatory network. Their spatial positioning is largely related to chemokine-mediated migration processes, which contribute to the remarkable heterogeneity of Tfh cells. Based on spatial localization, Tfh cells can be classified into GC-Tfh, FM-Tfh, and cTfh. Tph and mucosal Tfh-like cells may also be incorporated into this model due to their close relationship with conventional Tfh cells. Based on their close relationship with conventional Tfh cells, we propose an integrated system in which tissue-resident Tph and mucosal Tph constitute a tissue-resident pool, cTph and cTfh represent a circulating pool, and GC/FM-Tfh form a central pool. cTfh have been shown to be memory-pool, whereas cTph/Tph exhibit a memory phenotype. This suggests that cTfh may comprise the Tcm, Tem, and Tscm compartments of the Tfh lineage, while Tph/cTph correspond to its Trm pool. Thus, the memory pool can be considered an additional dimension—distinct from spatial localization and lineage commitment—consisting of memory states, with lineage subsets skewed toward particular memory phenotypes according to their different activating states.

We propose that the control of Tfh lineage commitment can be divided into two components: an imprinted part, wherein Tfh subsets may originate from other Th lineages (e.g., Tfh17 from Th17 and nTfr from Treg), and a *de novo* part orchestrated by BCL6-BLIMP1 balance (3). The generation of a Tfh subset with specific lineage features can be viewed as the summation of both imprinted and *de novo* contributions. That is, imprinted features initiate the expression of specific transcription factors, which shape initial differences in cytokine receptor profiles that ultimately manifest as major distinctions among Tfh subsets. These differences imply that variation in the microenvironment plays a critical role, reciprocally influencing the expression patterns of transcription factors, cytokines, and their receptors. Therefore, defining subsets based on surface markers, transcription factors, cytokines, and function remains a necessary foundation for clinical translation. High-dimensional technologies such as single-cell RNA sequencing and Cytometry by Time of Flight (CyTOF)—utilizing unsupervised clustering—could provide a greater panel of signatures utilized for machine learning to distinguish Tfh subsets more accurately (72).

However, to better capture the dynamics and plasticity of these states, which arise from variation in the imprinted and *de novo* components, non-clustering methods such as consensus non-negative matrix factorization (cNMF) are essential for modeling gene expression programs independent of limited marker sets. Pseudotime and trajectory analysis are valuable for inferring cellular dynamics, and single-cell TCR sequencing can elucidate clonal relationships among Tfh subsets.

The classification of Tfh cells retains clinical utility—for example, in assessing disease activity, severity, and therapeutic response—particularly in biopsy samples, where microenvironmental variation is limited. Caution is advised when interpreting results from blood samples, however, as circulating subset dynamics reflect the integrated immune state across multiple target organs. Defining Tfh subsets helps identify skewed states and recognize potentially pathological cell types, such as cTfh1 and Th1-skewed Tph, offering deeper immunological insight into diseases and therapies (such as selective cytokine targeting and JAK targeting therapies) and enabling more precise therapies. Current checkpoint blockade strategies targeting Tfh primarily focus on PD-1 and IL-21, and the insight into Tfh subsets might provide new targets. It may be beneficial to develop therapies that target skewing mechanisms, such as T-bet in Th1-oriented Tfh/Tph and atypical memory B cells in SLE. Elucidating Tfh memory states and their skewing could also improve our understanding of disease persistence, as in long COVID and other refractory or recurrent diseases, in which CAR-T therapies targeting the pathological memory cell subset might be beneficial.

In conclusion, defining Tfh heterogeneity extends beyond frequency-based cohort studies. A systemic understanding of relatively stable Tfh state and the dynamics of their development and plasticity will be crucial for unraveling disease mechanisms and ultimately guiding the development of more precise therapeutic interventions.
